# Dynamics of the formation of a hydrogel by a pathogenic amyloid peptide: islet amyloid polypeptide

**DOI:** 10.1038/srep32124

**Published:** 2016-08-18

**Authors:** Létitia Jean, Chiu Fan Lee, Peter Hodder, Nick Hawkins, David J. Vaux

**Affiliations:** 1 Sir William Dunn School of Pathology, University of Oxford, Oxford OX1 3RE, UK; 2 Department of Bioengineering, Imperial College London, South Kensington Campus, London SW7 2AZ, UK; 3 TA Instruments, Elstree WD6 3SZ, UK; 4 Department of Zoology, University of Oxford, Oxford OX1 3PS, UK

## Abstract

Many chronic degenerative diseases result from aggregation of misfolded polypeptides to form amyloids. Many amyloidogenic polypeptides are surfactants and their assembly can be catalysed by hydrophobic-hydrophilic interfaces (an air-water interface *in-vitro* or membranes *in-vivo*). We recently demonstrated the specificity of surface-induced amyloidogenesis but the mechanisms of amyloidogenesis and more specifically of adsorption at hydrophobic-hydrophilic interfaces remain poorly understood. Thus, it is critical to determine how amyloidogenic polypeptides behave at interfaces. Here we used surface tensiometry, rheology and electron microscopy to demonstrate the complex dynamics of gelation by full-length human islet amyloid polypeptide (involved in type II diabetes) both in the bulk solution and at hydrophobic-hydrophilic interfaces (air-water interface and phospholipids). We show that the hydrogel consists of a 3D supramolecular network of fibrils. We also assessed the role of solvation and dissected the evolution over time of the assembly processes. Amyloid gelation could have important pathological consequences for membrane integrity and cellular functions.

The deposition of misfolded and aggregated polypeptides to form amyloids is the hallmark of an increasing number of degenerative diseases including Alzheimer’s disease (AD) and non-insulin dependent diabetes mellitus type II (NIDDM)^1^. As human life expectancy increases, the burden of these diseases on societies is far reaching. Thus, it is paramount that the fundamental mechanisms of amyloidogenesis are understood. Although amyloid diseases are the consequences of the misfolding of many different polypeptides, they all result from polypeptides undergoing a conformational change to adopt a cross-β sheet structure, aggregating and forming fibrils^1^. The main cause of NIDDM is the loss of pancreatic β-cell mass and function, which has been linked to the cytotoxic effects caused by the aggregation of islet amyloid polypeptide (IAPP)^2^. IAPP is a 37-amino acid hormone co-secreted with insulin by the β-cells of the islets of Langerhans. Thus to fully understand the pathology associated with NIDDM, it is critical to elucidate the parameters influencing the kinetics of IAPP amyloid formation, and the role of cellular membranes in this process.

Amyloidogenesis occurs via a nucleation-dependent polymerisation process, during which the rate-limiting step is the formation of nuclei due to an energetically unfavourable association of monomers^3^. This results in a period known as the lag phase, after which fibrils grow at the expense of monomers (elongation) until the monomer concentration falls to the critical fibrillar concentration (the minimum monomer concentration required to form fibrils)^4^. Then fibril extension ceases and the reaction reaches its plateau phase. One consequence of this growth regime is that nucleation can be catalysed either by the addition of preformed aggregates or by hydrophobic-hydrophilic interfaces (HHIs).

Amyloidogenic polypeptides are surfactants due to their amphiphilicity^5^,^6^,^7^. Surfactants accumulate preferentially at HHIs, with their hydrophobic moieties shielded from the aqueous environment by interacting with the hydrophobic phase (e.g. air, phospholipids fatty acyl chains) and their hydrophilic moieties orientated toward the hydrophilic phase (e.g. aqueous environment, hydrophilic phospholipid head groups). Therefore, HHIs promote amyloidogenesis by concentrating amyloid precursors, promoting peptide chains alignment and favouring extended β-strand formation^5^,^8^. The adsorption to and accumulation of amyloid species on HHIs, and in particular biological membranes, is currently thought to be the cause of their toxicity due to membrane disruption (e.g. membrane thinning, altered membrane permeability, pore formation)^9^,^10^. Thus, it is essential to determine how amyloidogenic polypeptides behave at interfaces.

The *in-vitro* amyloidogenesis of IAPP and the AD amyloid-β peptide (Aβ) can be significantly promoted by an air-water interface (AWI; polar aqueous solution and non-polar gas)^7^,^11^,^12^,^13^,^14^. Therefore surface-induced amyloid fibrillisation is clearly different from that occurring in bulk solution. Furthermore, we recently demonstrated that the catalytic effect of lipids on IAPP assembly had previously been underestimated *in-vitro* due to a dominant AWI effect^7^. This demonstrates that the thermodynamic implications of the presence of an AWI on amyloid kinetics should be systematically considered *in-vitro*. Although the AWI is not as complex as membranes, it represents a very attractive HHI model due to its reproducibility and homogeneity. Thus, the dissection of the interfacial behaviour of amyloidogenic polypeptides can be achieved by using model HHIs such as the AWI. In turn, such studies can pave the way for investigations using more physiological and complex HHIs (e.g. biological membranes).

Many proteins adsorb at interfaces and form there multi-layered proteinaceous networks, which can be stabilised by gel formation. The gel layer itself can be stabilised by numerous non-covalent interactions to form a meshwork of aggregates (e.g. fibres), typical of amyloidogenic polypeptides. Gel formation has been reported for a range of classical amyloid or amyloid-like polypeptides, e.g. IAPP, α-synuclein and the RNA-binding protein fused in sarcoma^4^,^15^,^16^,^17^,^18^,^19^. However, to date the viscoelastic properties associated with gelation have only been fully characterised for non-pathological amyloidogenic polypeptides (e.g. β-lactoglobulin, insulin and spider silk), amphiphilic polypeptides forming layers at HHI (e.g. class II hydrophobins) and for fragments of pathological amyloidogenic polypeptides^15^,^20^,^21^,^22^,^23^,^24^,^25^,^26^. Such gel formation by amyloid species on the surface of cellular membranes would have huge consequences for membrane integrity and cellular functions.

In this paper, we investigated the physical properties of human IAPP assembly over time, along with its adsorption parameters in order to characterise key events that drive amyloid assembly. We studied IAPP adsorption at HHIs (both the AWI and physiologically relevant phospholipids) using surface tension measurements, its viscoelastic properties using rheology (both interfacial and bulk) and its morphology using electron microscopy. Moreover, we assessed the role of solvation in both fibrillisation and gelation, showing that the kinetics of elongation of amyloid precursors and of gel formation can be dependent on IAPP interactions with solvent water. We also characterised the viscoelastic properties of a hydrogel formed by the AD Aβ_1–40_, suggesting that viscoelasticity might be a generic property of amyloid peptides. To our knowledge this is the first full rheological characterisation of the dynamics of gelation by a full-length non-mutated pathological amyloid-forming polypeptide. This study serves as a stepping stone to understand the significance of the adsorption of amyloidogenic polypeptides at HHIs in a more physiological context, i.e. cellular membranes.

## Results

### IAPP assembles into a 3D supramolecular network of condensed fibrils

**Figure 1:**
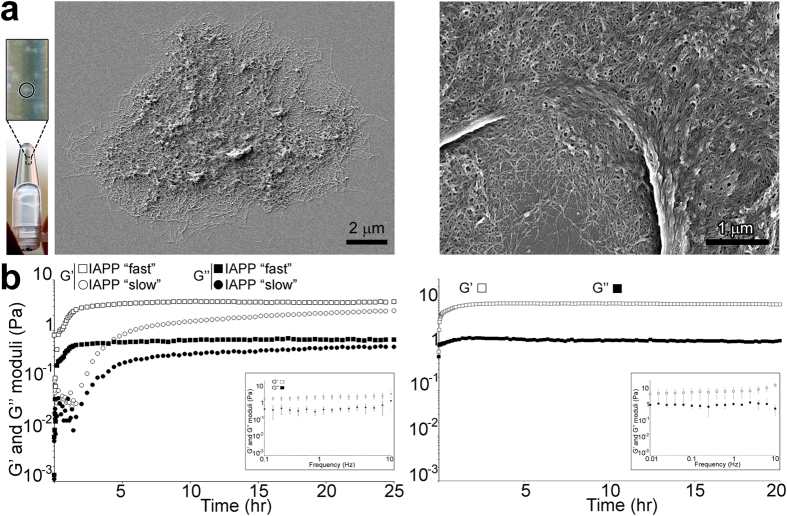
IAPP and Aβ form 3D hydrogels. (**a**, left panel) A 51.3 μM IAPP solution forms a gel, which contains macroscopic aggregates (*inset*; one example of an aggregate is circled). (*middle and right panels*) Morphological characterisation of the gel aggregates using SEM. (**b**) Rheological properties of a solution of 4 μM IAPP (*left panel*) or 30 μM Aβ (*right panel*) in water were assessed at 25 °C with a controlled displacement of 5 × 10^−3^ rads and a frequency of 0.5 Hz. Dynamic moduli G’ and G” as a function of time, with the *insets* showing G’ and G” as a function of angular frequency ω. The mean of at least three independent assays per curve is shown. Error bars represent ± SEM.

An IAPP-based hexapeptide (NL6) was previously shown to form a hydrogel composed of fibrillar networks^25^. Here, we assessed whether full-length IAPP behaves similarly. At concentrations typically used *in-vitro* to study IAPP (e.g. 4 μM), gelation had occurred after 24 hrs since inverting the tube resulted in the solution remaining at the bottom of the tube instead of falling into the cap. At 51.3 μM IAPP, macroscopic aggregates were visible in the gelled solution (Fig. 1a, *left panel*). The gel formed was quite fragile as it could be broken up by pipetting or shaking of the tube, as was previously observed for gels formed by tau_2–19_ and an Aβ_16–20_^23^,^27^. We characterised the morphology of the gel aggregates using scanning electron microscopy (SEM). IAPP clearly formed a 3D supramolecular fibrillar network (Fig. 1a*, middle panel*). At higher magnification, individual amyloid fibrils, amyloid fibril bundles as well as supramolecular networks of condensed amyloid fibrils were clearly identifiable (Fig. 1a, *right panel*).

### IAPP forms a 3D hydrogel

We then characterised the viscoelastic properties of the IAPP gel by oscillatory rheology. Typically gels display a non-zero shear elasticity. Strain amplitude measurements were performed to establish the linear viscoelastic region (Supplementary Fig. 1a *right panel*). Subsequently, an oscillation displacement of 5 × 10^−3^ rads was selected to ensure that the moduli were independent of strain. We also ascertained that DMSO (the IAPP solvent) did not affect the measurements (Supplementary Fig. 1a *left panel*). From the start of the experiment, a sol-gel transition (G’ = G”) of 4 μM IAPP could be observed (Fig. 1b*, left panel*). Rapidly the storage modulus G’ (a measure of surface elasticity) dominated over the loss modulus G” (a measure of surface viscosity), indicating elastic and solid-like behaviour, i.e. rapid gelation of the IAPP solution. A distinct cross-over point of G’ and G” could not be observed. A frequency sweep performed at the end of the experiment showed that G’ dominated G” at all frequencies tested with the Tan value (loss tangent, G”/G’) between 0.14 and 0.38 (Fig. 1b*, inset in left panel*). Both moduli also showed frequency independence. Altogether, these results are characteristic of the formation of a 3D hydrogel by the entire IAPP solution (bulk and AWI) resulting from a multilayer network structure with viscoelastic gel-like behaviour.

Formation of a 3D hydrogel was also observed for the full-length AD Aβ_1–40_, for which the sol-gel transition started immediately with G’ dominating G” (also G’ > G” for all frequency tested at the end of the experiment, with the Tan value < 1)(Fig. 1b, *right panel*). Both moduli also showed frequency independence. G’ reached plateau showing that gelation was complete at the end of our experiment.

### The complex dynamics of IAPP 3D hydrogel formation

The gelation of IAPP showed complex dynamics with two distinct kinetic regimes identified from 6 independent experiments; a “slow” regime (3 independent experiments) taking ~2 hrs for both moduli to increase at rates of 0.28 (G’) and 0.08 (G”) Pa/hr, and a “fast” regime (3 independent experiments) taking ~0.5 hr with rates of 4.7 (G’) and 2.3 (G”) Pa/hr (Fig. 1b *left panel* and 2*b*; Supplementary Figs 2a and 3a). For both regimes, G’ reached the same plateau value (~3.6 Pa) with the “fast” regime reaching it quicker than the “slow” regime (~3 and 22 hrs respectively), which suggests that equilibrium was reached. These data indicate that, irrespective of the kinetic regime, the bulk of the gelation process had occurred at the end of our experiment.

### Properties of the IAPP gel network, role of solvent molecules

To study IAPP gelation in more detail, we substituted H_2_O for deuterium oxide (D_2_O). Deuterium atoms are twice as heavy as hydrogen atoms, therefore D_2_O is a less reactive molecule than H_2_O and deuterium bonds are stronger than hydrogen bonds. Exchanging H_2_O for D_2_O allows probing for importance of molecular interactions in aqueous solution.

**Figure 2:**
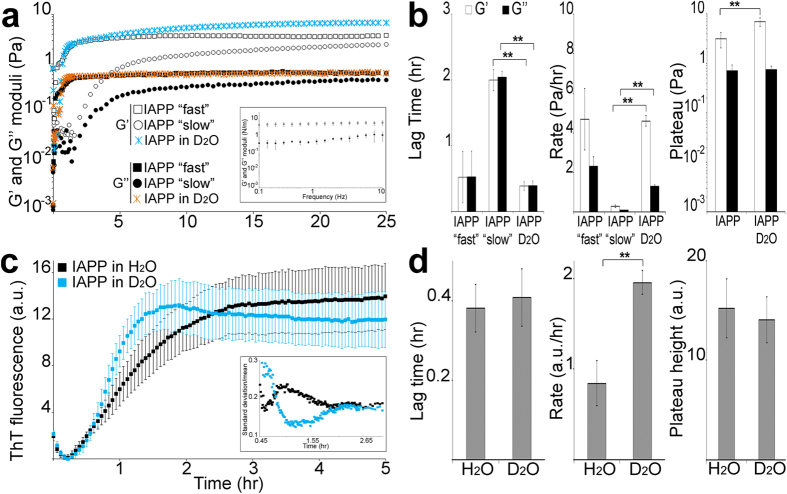
Role of solvent molecules in IAPP fibrillisation and gelation. (**a**) Rheological properties of a 4 μM IAPP solution in H_2_O or D_2_O were assessed at 25 °C with a controlled displacement of 5 × 10^−3^ rads and a frequency of 0.5 Hz. Dynamic moduli G’ and G” as a function of time, with the *inset* showing G’ and G” as a function of angular frequency ω. (**b**) The lag time for moduli increase, rate of increase and plateau reached are depicted. The mean of at least three independent assays is shown. Error bars represent ± SEM. (**c**) 4 μM IAPP was incubated in PBS with 32 μM ThT in H_2_O or D_2_O. ThT fluorescence changes were measured over time. a. u.: arbitrary units. The mean of at least three independent assays is shown. Error bars represent ± SEM. The *inset* shows the coefficient of variation (standard deviation over mean) as a function of time from the end of the lag phase up to beginning of plateau, i.e. to depict the variation over the elongation between the reactions in H_2_O and D_2_O. (**d**) The lag time, rate of increase and plateau reached are depicted. The mean of at least three independent assays is shown. Error bars represent ± SEM. **p < 0.005.

In D_2_O, the kinetic variations for IAPP gelation were abolished. The system adopted a unique regime taking ~0.4 hr for both moduli to increase at rates of 4.6 (G’) and 1.3 (G”) Pa/hr (Fig. 2a,b). The lag time for onset of gelation and the rate of moduli increase were not significantly different to that of the “fast” regime in H_2_O (Supplementary Fig. 2a). G’ reached a plateau (~7.0 Pa), indicating that the bulk of the gelation process had occurred, and this plateau was ~1.9 fold higher than that in H_2_O. G” plateau was unchanged by the isotopic substitution (~0.75 Pa). G’ dominated G” at all frequencies tested with the Tan value between 0.07 and 0.21 and moduli again showing frequency independence (Supplementary Fig. 1b). Our results indicate that a stronger and more rigid gel formed more rapidly in D_2_O than in H_2_O.

### Role of solvent molecules in IAPP fibrillisation

With D_2_O abolishing the kinetic variation of IAPP gelation, we turned our attention to aggregation stages preceding gelation in order to determine if D_2_O was solely affecting gelation or if the effect was more widespread. For this we substituted H_2_O for D_2_O in fibrillisation experiments, during which we followed the formation of stacked β-sheet amyloid structures by measuring the changes in fluorescence properties (emission from 445 to 482 nm) of the classical amyloid dye, thioflavin T (ThT)^28^.

Substituting H_2_O for D_2_O did not affect IAPP lag phase or plateau height (Fig. 2c,d). However, the elongation rate was significantly quicker (2.3 fold increase) and more importantly the variation in kinetics was reduced (Fig. 2c *inset*). Indeed, the coefficient of variation (standard deviation/mean) clearly shows that the elongation for the reactions in H_2_O is more variable than that of reactions in D_2_O. Thus, it is also clear that D_2_O reduced the variation in the elongation kinetics. Furthermore, the morphology of the species at the end of the fibrillisation reactions (i.e. when plateau was reached) was not discernibly different between H_2_O and D_2_O (Supplementary Fig. 4). Both reactions showed extensive clumps of fibrillar species (*left panels*) with sections of individual fibrils visible (*arrowheads right panels*).

### IAPP recruitment to the AWI

**Figure 3:**
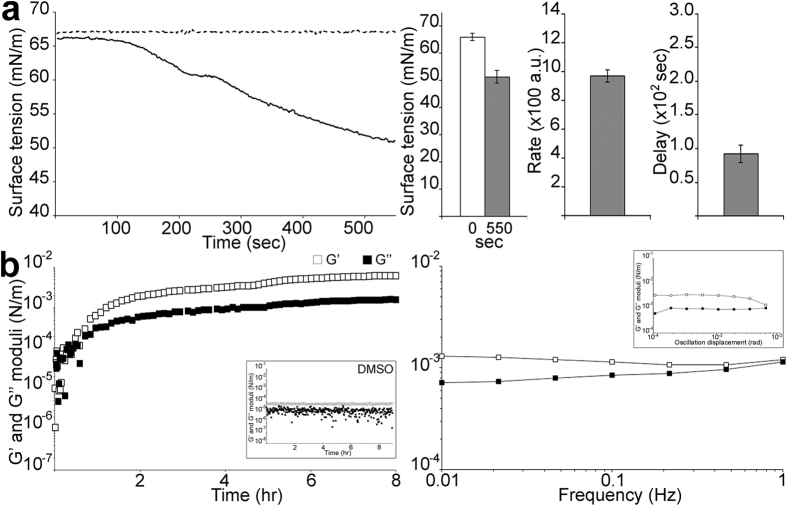
IAPP is surface active and forms a hydrogel at the AWI. (**a**) Dynamic measurement of the surface tension for 4 μM IAPP (black line) and for DMSO in water (the same amount as in the 4 μM IAPP)(black dotted line) was monitored over time (4 point moving averages of the raw data, *left panel*), with the surface tension of 4 μM IAPP at the start of the reaction and at 550 sec, the rate of decrease in surface tension and the time required for this decrease (*‘delay’*) depicted (*right panel*). a.u.: arbitrary units. The mean of at least three independent assays is shown. Error bars represent ± SEM. (**b**) Interfacial rheological properties of a 4 μM IAPP solution were assessed at 25 °C with a controlled displacement of 5 × 10^−3^ rads and a frequency of 0.5 Hz. (*left panel*) Dynamic interfacial moduli G’ and G” as a function of time, with the *inset* showing a DMSO control. (*right panel*) G’ and G” as a function of angular frequency ω. *Inset* shows G’ and G” as a function of oscillation displacement.

We then assessed IAPP adsorption to the AWI by measuring surface activity (Fig. 3a). IAPP lowered the surface tension of water from 66.0 ± 1.4 (at 0 sec) to 51.3 ± 2.3 mN/m (at 550 sec), with a mean delay of 92.6 sec for the progressive decrease in surface tension. Although the lowering of surface tension tended towards a plateau, it was not reached at 550 sec. Longer measurements were not possible as any significant evaporation of the drop would have influenced its shape and therefore surface tension. We previously used a surrogate measure of surface tension, which showed that 4 μM IAPP takes up to 2 hours to lower surface tension to a stable plateaued value^29^.

### IAPP forms a physical gel at the AWI

Since amyloidogenesis can be significantly promoted by the AWI, we next characterised IAPP gelation at the AWI by interfacial shear rheology. This technique characterises the viscoelastic properties of proteins adsorbed at interfaces and can provide important detail on the structure of the interfacial layer. The linear viscoelastic region was established (Fig. 3b, *inset in right panel*) and an oscillation displacement of 5 × 10^−3^ rads was then used. We also ascertained that DMSO (the IAPP solvent) did not affect the measurements (Fig. 3b*, inset in left panel*). The sol-gel transition (G’=G”) and gelation (G’ > G”) of 4 μM IAPP at the AWI were rapid (~0.5 hr)(Fig. 3b*, left panel*). By 2 hrs, G’ started plateauing but the two moduli were still slightly increasing indicating that interfacial gelation had occurred but equilibrium was not reached at the end of the experiment. G’ values after 8 hrs were ~6.2 mN/m, which is in the range previously reported for interfacial gelation of β-casein, β-lactoglobulin and uropathogenic *E. coli*^30^,^31^,^32^. A frequency sweep performed at the end of the experiment showed that G’ dominated G” at all frequencies tested, with the Tan value lower than 1 (Fig. 3b*, right panel*). Both moduli also showed frequency independence. Altogether, these results are typical of the formation by IAPP of a hydrogel at the AWI, and its evolution for 8 hrs.

### Role of HHI in IAPP gelation

Having established that IAPP can form a hydrogel both in the bulk solution and at the AWI, we investigated in more detail gelation by using physiologically relevant HHI in the form of 100 nm diameter liposomes. We used a 7:3 molar ratio of zwitterionic dioleoylphosphatidylcholine (DOPC) and anionic dioleoylphosphatidylglycerol (DOPG), which represents the minimum membrane charge density allowing complete IAPP binding to lipids but also resembles the lipid ratio of the pancreatic islet cell membrane^33^,^34^. The lipid concentration was equimolar to that of IAPP and the liposome addition increased the HHI surface area by ~4 when compared to just having the AWI present. Not only had this approach allowed us to investigate the role of *in-vivo* HHI but also to assess the importance of HHI on gelation.

**Figure 4:**
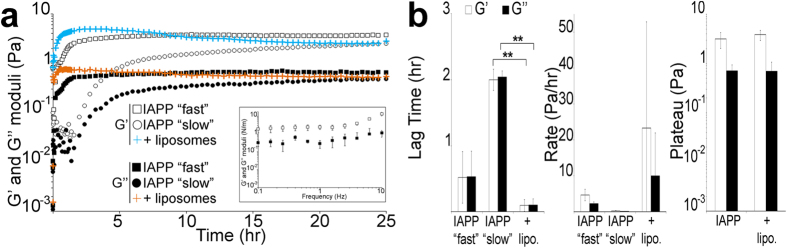
Role of HHI in IAPP gelation. (**a**) Rheological properties of a 4 μM IAPP solution in H_2_O in presence of 4 μM liposomes (7 DOPC:3 DOPG) were assessed at 25 °C with a controlled displacement of 5 × 10^−3^ rads and a frequency of 0.5 Hz. Dynamic moduli G’ and G” as a function of time, with the *inset* showing G’ and G” as a function of angular frequency ω. (**b**) The lag time for moduli increase, rate of increase and plateau reached are depicted. The mean of five independent assays is shown. Error bars represent ± SEM. **p < 0.0006. lipo.: liposomes.

We first ascertained that the liposomes did not affect the measurements (Supplementary Fig. 1C). Gelation of 4 μM IAPP in presence of 4 μM liposomes (5 independent experiments) adopted a unique regime (no kinetic variation) that was even faster than the “fast” regime in absence of liposomes (~0.1 versus ~0.5 hr in lag time and 23.4 versus 4.7 Pa/hr for the rate)(Fig. 4 and Supplementary Figs 2b and 3b). G’ reached a plateau (~3.8 Pa), similar to that in absence of liposomes, indicating that the bulk of the gelation process had occurred and that the presence of liposomes did not change the gelation equilibrium. A frequency sweep performed at the end of the experiment showed that G’ dominated G” at all frequencies tested, with the Tan value lower than 1 (Fig. 4a*, left panel*). Both moduli also showed frequency independence. These results indicate that gelation critically depends on HHIs.

## Discussion

Adsorption of amphiphilic amyloidogenic polypeptides to HHIs catalyses amyloidogenesis by increasing the effective peptide concentration and promoting β-sheet formation^5^,^7^,^11^,^12^,^14^. The goal of this study was to investigate the evolution of early key events that drive amyloidogenesis at HHIs. Human IAPP was used as an amyloid system as it is critical to elucidate the mechanism of its assembly to fully understand its role in NIDDM. Despite previous reports of IAPP aggregate formation in a gel and gel formation of IAPP_20–29_, the viscoelastic properties of the gelation process have never been investigated^15^,^16^,^17^.

We have clearly showed gelation of IAPP both in the bulk solution and at HHIs, both the *in-vitro* AWI and physiological lipids. We have also demonstrated that IAPP forms a 3D supramolecular network of condensed amyloid fibrils and we believe these to be responsible for the gelation process. Thus, we propose that IAPP gel network would be non-covalently cross-linked through typical amyloid β-sheet interactions (e.g. hydrogen bonds, electrostatic and hydrophobic interactions). We believe it is unlikely that gelation resulted only from fibril entanglement as substitution of H_2_O by D_2_O showed an effect on the kinetic regime, suggesting that at least hydrogen-bonding plays a role. Furthermore, it has been shown that gelation of β-lactoglobulin resulted from hydrophobic and van der Waals interactions between fibrils^21^. Similarly, gelation of whey proteins was shown not to be via entanglements but through non-covalent interactions between aggregates^35^. Therefore the IAPP network must be classified as a 3D ‘physical’ gel. Indeed, non-covalently cross-linked physical gels are commonly formed from the association of supra-or supermolecular aggregates such as amyloid fibrils, as we observed^21^,^22^,^31^.

Our rheological data suggest gelation is the natural final state of amyloidogenesis under our experimental conditions. However in H_2_O, we clearly observed distinct kinetic regimes, “slow” and “fast”, which collapse into a single “fast” regime in D_2_O. We believe these variations in the speed of gelation to be genuine kinetic variations rather than arising from sample-to-sample variations between independent measurements, often typical of amyloid studies. Indeed, sample-to-sample variations would have generated a range of kinetics covering the spectrum between what we called the “slow” and “fast” regimes (i.e. curves spanning the all range) rather than two distinct groupings, as we observed. The observed kinetic variation suggests the existence of multiple gelation pathways. This is unexpected for a macroscopic system in which the self-averaging effects of a large number of molecules should mask distinct kinetic rates^36^.

What might explain the macroscopic stochastic variation we observed for IAPP gelation would be the system separating into phases? We hypothesise that the variation may result from phase separation, during which IAPP-rich droplets form due to hydrophobic interactions (Supplementary Fig. 5). Phase separation has previously been shown to play a role cellularly, but more importantly to enhance amyloid-like fibrillisation by increasing the propensity of nucleation events within the droplets and to lead to formation of fibrillar-based hydrogels^19^,^37^,^38^,^39^,^40^. Due to the finite size of the system and after a transient period, only a few droplets should co-exist in the system, with gelation occurring slowly within each. As fibrils grow, so do the droplets and the gel eventually percolates the whole system. In our hypothesis, slow and fast pathways could represent the rheological measurements of a one-droplet and two-droplet systems respectively during the transient period. Our gelation data in D_2_O (see below), showing a unique regime similar to the “fast” one in H_2_O without any kinetic variation, also support our hypothesis of IAPP phase separation preceding gelation. Indeed, phase separation kinetics of deutereous polymer have previously been shown to be faster than that of hydrogenous polymer^41^. Faster phase separation should trigger faster gelation kinetics, as we observed in D_2_O. Protein solubility was also shown to be lower in D_2_O due a stronger hydrophobic effect (i.e. a stronger tendency for proteins to associate), which would be suggestive of the system separating into phases^42^,^43^. A D_2_O triggered decrease in protein solubility (or increase in aggregation) would also be consistent with a more favoured phase separation (i.e. initiated more quickly and the phase separated drops growing more rapidly and thus eliminating the co-existence of multiple drops in the system). In a finite system, this can lead to a decrease in stochastic variations, which may explain why the separate “fast” and “slow” kinetic regimes are not observed in D_2_O. To ascertain whether IAPP gelation occurs through phase separation would be worthy of further investigations that are beyond the scope of this study.

A gel formed in an aqueous environment is called a hydrogel. Hydrogel are vulnerable to shear thinning by application of a force (e.g. pipetting), which decreases gel viscosity and forces the physical crosslinks to reorganise. These characteristics were met by the IAPP gel. In a hydrogel, water participates in hydrogen bonding through bridging of charged groups and acts as a bridging element maintaining gel structure. Thus to address the properties of the gel network, we substituted H_2_O for D_2_O in rheology experiments. No significant change in gel viscosity was observed in D_2_O, with the G” plateau identical to that in H_2_O. Importantly, we found an increase in viscoelastic properties with the G’ plateau nearly twice higher than that in H_2_O, indicating that a stronger and more rigid gel had formed. A similar rigidity and strength for gels in D_2_O has previously been observed for gelatin and κ-carrageenan, which was attributed to stronger intermolecular attractions in D_2_O, leading to an increase density of packing and a more extensive gel network^41^,^42^,^44^. In D_2_O, the critical micellar concentration was also shown to be lower and therefore the rate of aggregation to be faster, leading to a higher number of aggregates at the end of gelation^42^,^43^. Indeed, we observed faster kinetic rate for IAPP elongation in D_2_O (see below), which in turn should lead to a faster and less variable kinetic of gelation. Indeed, our results suggest that the effect of D_2_O in accelerating kinetic and reducing variation is widespread as D_2_O affected both early (the elongation rate of fibrillisation) and late (gelation) stages. Our results imply that, during rapid gelation (“fast” regime), a hydrogen bonding network between IAPP and H_2_O molecules is not the most relevant stabilisation factor and that hydrogen bonding between IAPP aggregates are more important. Conversely, slower gelation (“slow” regime) may partly be supported by hydrogen bonding absent in D_2_O.

We also investigated the importance of IAPP solvation in stages prior to gelation, i.e. fibrillisation. The main effect of D_2_O should be to weaken IAPP solvation and strengthen hydrophobic interactions, which should increase aggregation^42^,^43^. Indeed, we found that D_2_O accelerated the elongation rate of fibrillisation. The lack of effect of D_2_O on nucleation is not surprising as nuclei would mostly form from monomers adsorbed at the AWI and this would not depend on whether the solvent was H_2_O or D_2_O. This is consistent with the study by Chi *et al.* showing that Aβ assembly at the AWI is driven by hydrophobicity, and changes in solvent conditions (such as pH and ionic strength) had no effect^11^. Moreover, human IAPP monomers were found, by simulations, to adopt 3 main conformations, with β-hairpins dominating and being the least soluble and flexible, again suggesting that changing solvent should not strongly affect free monomer conformation^45^. However, elongating species would start to extend into the bulk solution and would become dependent on solvent. Studies proposed that fibril elongation occurs predominantly via lateral binding to and diffusion of monomers on fibril sides before transfer to the growing ends, with direct docking onto fibril ends being a minor pathway^29^,^46^,^47^. Furthermore, the partially buried hydrophobic cluster of β2-microglobulin monomers was shown to become solvent exposed in laterally attached monomers^46^,^47^. Monomer binding laterally to fibrils would occur through a range of non-specific interactions but without the formation of a stable hydrogen-bond network, which may explain why elongation is sensitive to subtle changes in solvent conditions, as we observed by substituting H_2_O for D_2_O. A higher concentration of monomers on the fibril surface generally leads to higher elongation rate^29^. Furthermore, the effect of D_2_O should be more prevalent while the monomer concentration is high, like during elongation. Successful attachment of monomers to fibril ends would also require the solvent to be removed, which suggests that elongation should be slower in H_2_O than in D_2_O, as we indeed observed. Thus, D_2_O would increase hydrophobic interactions leading to faster monomer association onto fibrils and generally to faster elongation. Finally, the hydrophobic cluster of β2-microglobulin monomers was shown to become buried when monomers are transferred to fibril ends and before stabilisation occurs through hydrophobic and hydrogen interactions, which suggests that the system would again become solvent independent^46^,^47^. Indeed, fibrillisation is known to be accompanied by loss of bound water, with side-chain interdigitating to produce a tightly packed, dry, hydrophobic interface between β-sheet layers. The charged side-chains on the exterior would be fully solvated, which keeps fibrils soluble during gelation, preventing higher order assembly that would lead to precipitation^48^. This would explain why IAPP end stages (when plateau is reached) would be solvent independent (i.e. no effect of D_2_O).

**Figure 5:**
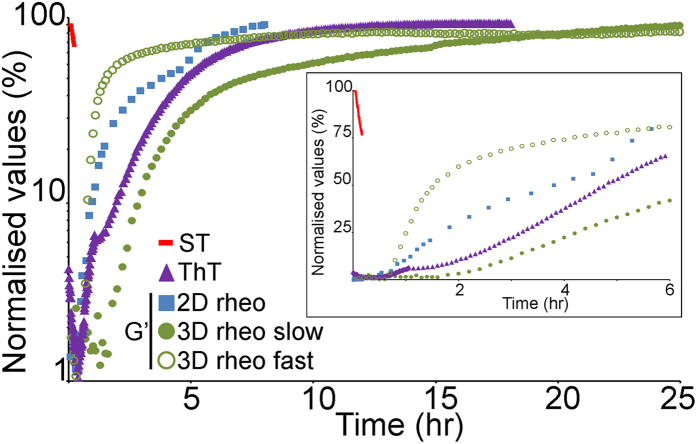
Evolution over time of the processes involved in IAPP supra-molecular assembly. In order to facilitate kinetic comparison, data has been summarised from the complete data set shown in other Figures. To be able to compare the kinetics between gelation and fibrillisation, the ThT fibrillisation assays (mean of 3 independent experiments) shown was performed at 25 °C, the same temperature as for the rheology experiments. Values have been normalised to the maximum value in each experiment and are expressed as a percentage of this value. For clarity only G’ is shown for gelation. The *inset* shows a zoom-in of the time axis. rheo stands for rheology, ST for surface tension.

Some striking features were revealed when we analysed the evolution over time of IAPP adsorption, fibrillisation and gelation (Fig. 5). Not only is it clear that full interfacial adsorption (i.e. when maximum lowering of surface tension is achieved) preceded any other phenomenon, but the onset of fibrillisation and gelation only occurred as soon as, or very shortly after, IAPP had adsorbed (*inset* of Fig. 5). The fact that the aging time required for the sol-gel transition at the AWI was longer (~0.5 hrs) than IAPP initial adsorption at the AWI (~93 sec), suggests that interfacial gelation did not occur as soon as IAPP adsorbed at the AWI. It is highly likely that molecular and structural rearrangements take place at the interface during gelation. We propose that once diffused to the AWI, IAPP monomers would have to undergo a conformational change to ‘orientate’ themselves with their hydrophobic side-chains pointing towards the air and the hydrophilic facing the water. Subsequently, further rearrangements would allow maximal 2D packing at the interface before lateral intermolecular interactions (e.g. β-sheet formation during fibrillisation and subsequent interfibrillar interactions) would occur to form an interfacial layer. These rearrangements should be affecting the rheological properties of the adsorbed layer. The first interfacial rheological changes were observed after ~0.5 hrs (G’ > G”), which suggests that IAPP adsorption and rearrangement at the AWI were rapid and sufficient to start changing the viscoelastic properties of the interface, e.g. formation of aggregates large enough to affect the interfacial rheology. This is supported by fibrillisation data (done at 25 °C the same temperature as the rheology experiments) showing that IAPP elongation, i.e. formation of aggregates larger than nuclei, started after a lag phase of 0.56 ± 0.11 hrs (Fig. 5). In the entire system (bulk and AWI, 3D rheology), the onset of gelation for the “fast” regime was concomitant with onset of elongation, whereas that of the “slow” regime occurred after onset of elongation (~2 hrs). Then both at the AWI and for the “slow” regime in the entire system, G’ started reaching a plateau, which indicates that most of the gelation had taken place, concomitantly with fibrillisation starting to plateau. Therefore, the aggregates may have created a large enough meshwork to fully behave as a gel. For the “fast” regime of gelation in the entire system, plateau was reached as fibrils were still elongating (around half way through the elongation of the fibrillisation assays).Thus, overall gelation is achieved during fibril extension or concomitantly to fibril extension ceasing. The surface tension was tending towards a plateau more rapidly than G’ did, indicating that any structural rearrangements occurring after initial adsorption affected the interfacial rheological properties without strongly influencing the surface tension. Once formed, the IAPP adsorbed layer(s) at the AWI and the assembly in the entire system remained elastic and solid-like, with G’ > G” and the loss tangent remaining lower than 1 over the range of frequencies tested. As both moduli were still increasing at the end of the 2D rheology experiment, it suggests that once gelation has started further structural rearrangements may be taking place at the AWI.

A detailed characterisation of the viscoelastic properties of the gelation process of a full-length pathological amyloidogenic polypeptide has never been undertaken before. Our study has clearly demonstrated the complex dynamics of the formation by IAPP of a 3D hydrogel, consisting of a 3D supramolecular network of condensed amyloid fibrils. We also showed the formation of a 2D physical gel at HHIs. Furthermore, we demonstrated the critical importance for gelation of the presence of an HHI, either AWI or physiological phospholipids, as increasing the HHI surface area accelerated the kinetics. We previously showed that both amyloid precursors (pre-fibrillar) and fibrillar species strongly adsorb at the AWI, with the fibrillar assembly adsorbing more strongly than precursors^4^. These results suggest that, once adsorbed, monomers aggregate at the AWI and that when formed fibrils remain at the AWI. Therefore, we hypothesise that gelation within the bulk solution (3D) may be initially catalysed by the formation of a 2D physical gel at HHIs. Our and other studies also suggest that gelation might be a generic property of conventional amyloid and amyloid-like polypeptides^4^,^15^,^16^,^17^,^18^,^19^,^20^,^21^,^22^,^23^,^24^,^25^,^26^. Loss of membrane integrity, leading to cell apoptosis, is currently thought to be the cause of toxicity by amyloid intermediates. This has been shown to occur via a variety of mechanisms, from carpeting to membrane thinning and pore formation^49^. However, gelation of amyloid species could also be a cause of toxicity. Gel formation within or outside cells could perturb membrane integrity as much as peptide adsorption, interfere with signalling pathways and cell motility, or alter the collagen gel network of the extracellular matrix. Tau gel has been postulated to intercalate into the cytomatrix, to interfere with the dynamic rearrangement of the actin microfilaments, which would lead to the production of intracellular stress forces and ultimately to the retraction of neuronal dentrites^27^. Although IAPP fibrils are deposited extracellularly in the pancreas, there is evidence that IAPP oligomers can form inside cells and do not need to be secreted^50^. Therefore, IAPP toxicity could result from membrane damage anywhere along the secretory pathway (from the endoplasmic reticulum to the plasma membrane).

## Methods

### Peptide preparation

Lyophilised synthetic human IAPP (Bachem) and Aβ_1–40_ (EZBiolab) were purchased already purified by reverse-phase high performance liquid chromatography. Both peptides were resuspended in DMSO (IAPP at 512.4 μM and Aβ_1–40_ at 1.6 mM), sonicated and centrifuged for 1 hr at 15,000 g at +4 °C prior to use (to remove any pre-aggregated species). DMSO was used to maintain the peptides in a monomeric pool lacking any β-sheet secondary structures^51^.

### Liposome preparation

Chloroform stocks of DOPC and DOPG (Avanti Polar Lipids) were mixed in a 7:3 molar ratio. After solvent evaporation and desiccation, the lipids were hydrated in water and extruded through 0.1 μm pore size filter (Whatman). The number of liposomes was assessed with a NanoSight LM10 apparatus (Malvern Instruments).

### Surface tensiometry

The surface tension of 4 μM IAPP (from the DMSO stock solution) in water was measured at 21.8 °C by pendant drop shape analysis using a ‘Tracker’ video enhanced drop tensiometer (ITConcept), as previously described^52^. At least three independent assays were performed, with each drop done in duplicate.

### SEM

51.3 μM IAPP (from the DMSO stock solution) in PBS in a 200 μl reaction volume was left to fibrilise at room temperature in a quartz cuvette. The macroscopic aggregates at the AWI were pipetted out, deposited on a coverslip and then on a sample holder using conductive tape, dehydrated with increasing percentages of ethanol, dried with hexamethyldisilazane, sputtered with ~7 nm gold before being examined with a Zeiss NVision 50 microscope using an accelerating voltage of 2 kV.

### Rheology

Measurements were performed, at 25 °C, on a Bohlin Gemini 200 HR Nano rheometer (Malvern Instruments). IAPP or Aβ_1–40_ (from the DMSO stock solution) was pipetted onto the lower plate of the rheometer and water or D_2_O (Sigma-Aldrich) was added to get a 1.4 ml reaction volume and a 4 μM IAPP or 30 μM Aβ_1–40_ final concentration. Liposomes at a 4 μM final concentration (as an initial lipid concentration adjusted according to the number of liposomes measured at the end of the preparation) were also added when required. Then the upper plate (measuring cone geometry, *D* = 40 mm, 4° incline) was lowered as slowly as possible onto the sample to ensure a completely filled gap. An environmental cuff with moistened tissue inside was placed around the geometry to prevent sample dehydration. Controls containing DMSO with or without liposomes were also performed. Oscillation time sweeps were recorded with a controlled displacement of 5 × 10^−3^ rads and a frequency of 0.5 Hz. Frequency sweeps were also performed. Statistical analysis was performed with the two-sample t-test. For IAPP in absence of liposomes, 6 independent experiments were performed; for IAPP in presence of liposomes, 5 independent experiments were performed.

### Fibrillisation

4 μM IAPP were dispensed in a 96-well plate (μclear, polystyrene, black wall, clear bottom; Greiner Bio-One) with 32 μM ThT in PBS, in a 100 μl final solution volume in H_2_O or D_2_O. ThT fluorescence (excitation 450 nm, emission 480 nm) was measured at 37 °C on a BMG Polarstar plate reader (BMG labtech) using a bottom-bottom configuration (optical fiber system detecting emission signal from the bottom of the well). The values of control wells (buffers without peptide) were subtracted from the values of test wells (peptide). The lag phase was obtained from the intercept on the time axis of the line formed tangent to the inflection point. The elongation rate was obtained from the slope at the inflection point of the sigmoidal curve and the plateau height from an average of the highest curve values attained at the end of the experiment. At least three independent assays, with at least 9 wells per replicate, were performed and analysed with the two-sample t-test.

### Interfacial rheology

Measurements were performed, at 25 °C, on a DHR-2 interfacial rheometer (TA Instruments) using a double wall ring and an 18 ml reaction volume. IAPP (from the DMSO stock solution) was pipetted at the bottom of the vessel and water was then added to give a 4 μM IAPP final concentration. Control containing DMSO was also performed. Oscillation time sweeps were recorded with a controlled displacement of 5 × 10^−3^ rads and a frequency of 0.5 Hz. Frequency sweeps were also performed.

## Additional Information

**How to cite this article**: Jean, L. *et al.* Dynamics of the formation of a hydrogel by a pathogenic amyloid peptide: islet amyloid polypeptide. *Sci. Rep.* **6**, 32124; doi: 10.1038/srep32124 (2016).

## Supplementary Material

Supplementary Information
