# Supplementary material for: Dynamics of the formation of a hydrogel by a pathogenic amyloid peptide: islet amyloid polypeptide
Source: Sci Rep. 2016 Aug 18;6:32124. doi: 10.1038/srep32124 (PMC4989184; doi:10.1038/srep32124)

Dynamics of the formation of a hydrogel by a pathogenic amyloid peptide: islet amyloid polypeptide

Létitia Jean, Chiu Fan Lee, Peter Hodder, Nick Hawkins and David J. Vaux

Supplementary information

Fig. 1. Rheological behaviour of DMSO in H_2O and linear viscoelastic region for IAPP in H_2O and D_2O. (*a*) Dynamic moduli G' and G'' as a function of time for DMSO in H_2O (*left panel*). G' and G'' as a function of oscillation displacement for a solution of 4 μM IAPP in H_2O (*right panel*). (*b*) G' and G'' as a function of oscillation displacement for a solution of 4 μM IAPP in D_2O. (*c*) Dynamic moduli G' and G'' as a function of time for 4 μM liposomes in H_2O.

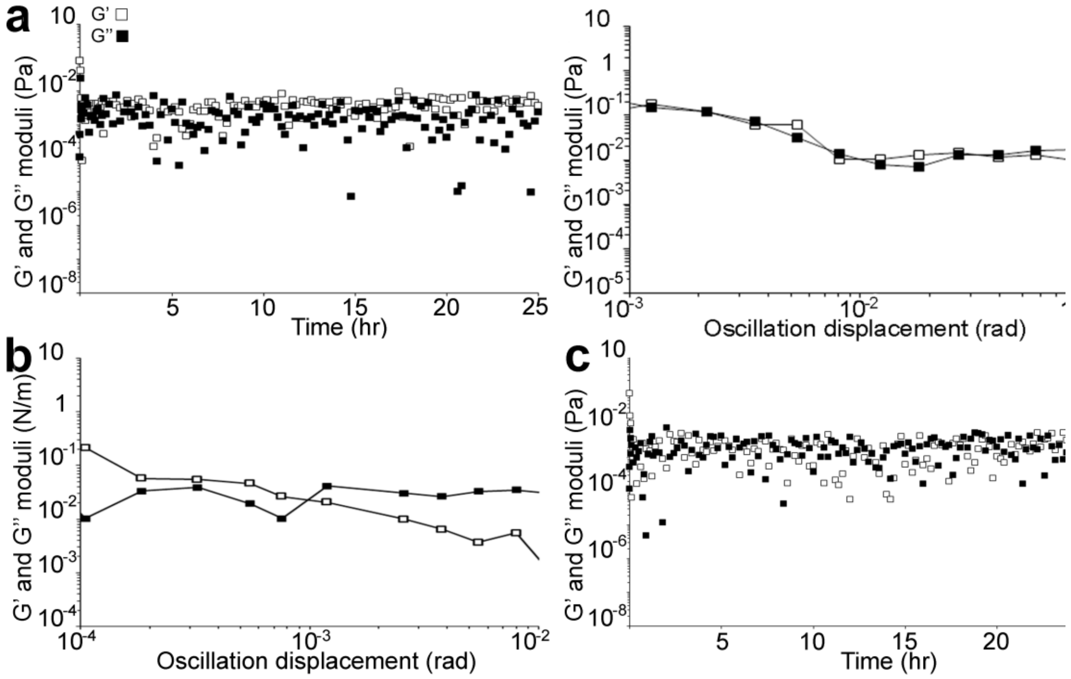

Fig. 2. Lag times of the gelation processes. (a) Zoom in on the lag phases of the rheology of a 4 µM IAPP solution in H_2O or D_2O. Dynamic moduli G' and G'' as a function of time are represented. (b) Zoom in on the lag phases of the rheology of a 4 µM IAPP solution in H_2O in presence of 4 µM liposomes (7 DOPC:3 DOPG). Dynamic moduli G' and G'' as a function of time are represented.

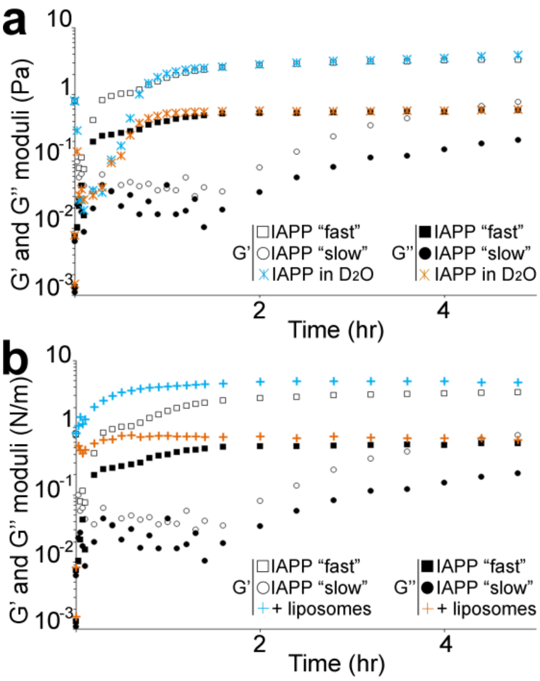

Fig. 3. IAPP forms a 3D hydrogel by following two distinct kinetic regimes, which formation is dependent on HHI. Rheological properties of a 4 µM IAPP solution in water, in absence (*a*) or presence (*b*) of 4 µM liposomes (7:3 ratio of DOPC to DOPG), were assessed at 25ºC with a controlled displacement of $5x10^{-3}$ rads and a frequency of 0.5 Hz. The dynamic modulus G' as a function of time is represented. The *right panel* represents a zoom in over the lag phase time. Each curve represents independent experiments (6 independent experiments in (*a*) for which 3 showed the fast regime and 3 showed the slow regime). The black curve in (*b*) represents 5 independent replicates in presence of liposomes.

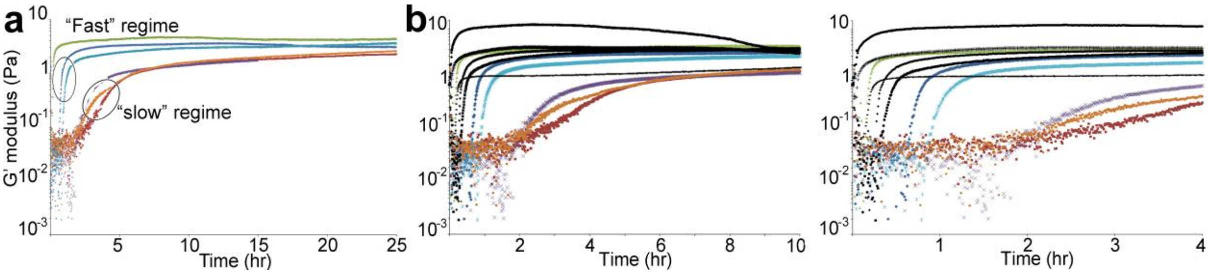

Fig. 4. Morphology of IAPP species from fibrilisation reactions in H_2O or D_2O. 4 μM IAPP was incubated in PBS with 32 μM ThT in H_2O (*a*) or D_2O (*b*) at 37°C Fibrillisation reactions were harvested at plateau (as measured by ThT fluorescence). The solutions were adsorbed onto 200 mesh carbon film copper grids, negatively stained with 2% aqueous uranyl acetate, washed with distilled water, air dried, and viewed with a Tecnai electron microscope (Philips). Examples of sections of individual fibrils are shown by arrowheads.

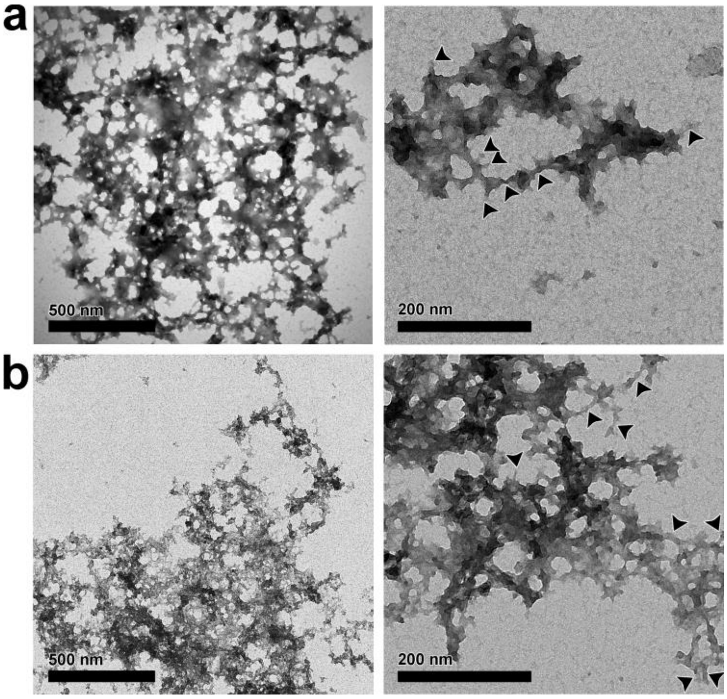

Fig. 5. Hypothetical model for how IAPP solution proceeds to become a gel. Starting from a solution of monomers, fibrillisation and phase separation of monomers and fibrils occur (potentially concurrently) and as a result, dispersed droplets of fibrils and monomers co-exist in the solution. The droplets then grow and coalescence, which leads to a depletion of droplets in the system. Gelation subsequently is initiated within the remaining droplets. Finally, the gelled droplets percolate the whole system.

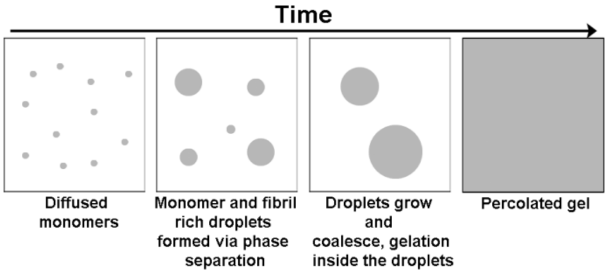

Supplement: Supplementary Information [file srep32124-s1.pdf]
